# Baseline Fusobacterium Abundance Predicts Ustekinumab Response in Crohn's Disease: A Prospective Microbiome Cohort Study

**DOI:** 10.1111/1751-7915.70250

**Published:** 2025-10-24

**Authors:** Chengran Wang, Yanping Hao, Yunyun Liu, Le He, Su Xu, Minna Zhang, Xin Wang, Honggang Wang

**Affiliations:** ^1^ Department of Gastroenterology The Affiliated Huaian No. 1 People's Hospital of Nanjing Medical University Huai'an China; ^2^ Department of Gastroenterology The Affiliated Yancheng First Hospital of Nanjing University Medical School Yancheng China; ^3^ Department of Gastroenterology The Affiliated Suqian First People's Hospital of Nanjing Medical University Suqian China; ^4^ Department of Gastroenterology, PhD The Affiliated Yancheng Chinese Medicine Hospital of Nanjing University of Chinese Medicine Yancheng China; ^5^ Department of Gastroenterology The Affiliated Hospital of Xuzhou Medical University Xuzhou China

**Keywords:** 16s rRNA, Crohn's disease, fusobacterium, ustekinumab

## Abstract

The gut microbiota composition in Crohn's disease (CD) patients may influence their response to ustekinumab (UST) therapy. A total of 51 patients with active CD undergoing UST therapy were prospectively enrolled. Clinical activity was evaluated using the Crohn's Disease Activity Index (CDAI), and faecal microbiota were characterised by 16S rRNA sequencing at baseline and week 24. Microbial compositional and functional alterations were assessed, and their correlations with clinical outcomes were examined. At week 24, 46.7% of patients achieved clinical remission and 82.2% achieved clinical response. At baseline, *Megamonas* (*p* = 0.009) and *Erysipelatoclostridium* (*p* = 0.030) were enriched in the remission group, whereas *Fusobacterium* (*p* = 0.016) was more abundant in the non‐remission group and correlated positively with C‐reactive protein (CRP) but negatively with body mass index (BMI) and serum albumin (ALB). Longitudinal analysis showed that CR patients exhibited increased *Clostridium sensu stricto 1* (*p* = 0.028) and decreased *Granulicatella* (*p* = 0.043) after 24 weeks. This study provides real‐world evidence supporting the clinical efficacy of UST in Asian patients with active CD. The observed association between elevated baseline *Fusobacterium* abundance and poorer treatment response suggests a potential microbial influence on therapeutic outcomes. These findings highlight the potential of *Fusobacterium* as a predictive biomarker for UST response and could provide a rationale for integrating microbiota‐modulating strategies to enhance the efficacy of biologics in the future.

## Graphical abstract

Baseline enrichment of *Fusobacterium* predicts poor response to ustekinumab in Crohn's disease, while *Megamonas* and *Erysipelatoclostridium* are enriched in responders, suggesting gut microbiota as a potential biomarker for personalised therapy.

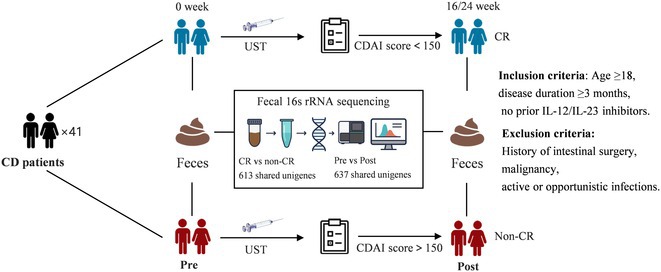

## 1 Introduction

Crohn's disease (CD) is a chronic inflammatory bowel disease (IBD) with rising incidence worldwide, including in developing countries like China (Hracs et al. 2025). Environmental shifts, urbanisation and lifestyle changes have contributed to this trend (Xu et al. 2023), while CD symptoms such as abdominal pain, fatigue and malnutrition severely impair quality of life (Holten et al. 2023).

Ustekinumab (UST), a monoclonal antibody targeting the shared p40 subunit of IL‐12 and IL‐23, is increasingly used for moderate‐to‐severe CD (Chaparro et al. 2022; Sandborn et al. 2022; Bokemeyer et al. 2025). Yet, clinical response remains variable, underscoring the need for reliable predictors of treatment efficacy (Bertani et al. 2024).

Gut microbiota play a key role in CD pathogenesis and may influence treatment outcomes (Shan et al. 2022; Iliev et al. 2025). *Fusobacterium* has previously been found enriched in the gut of CD patients and is thought to contribute to disease progression (El Mouzan et al. 2018). However, no study has yet linked *Fusobacterium* abundance to UST treatment response.

To our knowledge, this is the first study to investigate the association between baseline *Fusobacterium* abundance and the clinical efficacy of UST in Asian patients with CD. These findings provide novel insights into microbiota‐based patient stratification and the potential for personalised treatment strategies in IBD.

## 2 Methods

### 2.1 Study Design and Participants

This was a prospective, single‐center observational cohort conducted at the Affiliated Huaian No. 1 People's Hospital of Nanjing Medical University. Consecutive adults with established CD who initiated UST from 1 January to 31 December 2022, were enrolled according to a pre‐specified protocol. The inclusion criteria were as follows: age ≥ 18 years, a definite CD diagnosis ≥ 3 months, and no prior exposure to IL‐12/23 inhibitors. Exclusion criteria included a history of bowel surgery (major intestinal resection), active malignancy and current active opportunistic infections. Disease location and behaviour were classified according to the Montreal classification, and perianal disease was recorded separately.

All patients received weight‐based intravenous (IV) induction at week 0 (260 mg for body weight ≤ 55 kg; 390 mg for > 55 to ≤ 85 kg; 520 mg for > 85 kg), followed by 90 mg subcutaneous (SC) every 8 weeks per label. Patients were prospectively followed for 24 weeks with predefined assessment time points at week 0 and week 24 (visit window ±2 weeks). Clinical data and biospecimens were collected at each visit. Due to practical constraints, dietary history and recent antibiotic exposure were not collected, which may confound gut microbiota composition. Loss to follow‐up (LTFU) was defined as missing the week 24 visit outside the window; LTFU cases were excluded from the efficacy analysis, and reasons for LTFU were documented.

The study adhered to the ethical principles outlined in the Declaration of Helsinki. The study was approved by the Institutional Review Committee of the Institutional Review Board (IRB) of the Affiliated Huaian No. 1 People's Hospital of Nanjing Medical University (Approval ID: KY‐2023‐065‐01). Patients provided informed consent for microbiota data reuse.

### 2.2 Clinical Outcomes

Clinical disease activity was measured by the CD activity index (CDAI), evaluated at week 0 and week 24. Clinical activity was defined as CDAI ≥ 150, clinical remission as CDAI < 150, and clinical response as a reduction in CDAI ≥ 100 points from baseline. The primary outcome was the comparison of faecal microbiota composition in selected patients based on real‐world data.

### 2.3 Stool Sample Collection

Fresh faecal samples were collected from CD patients at week 0 (Pre) and week 24 (Post), stored at −80°C until 16S rRNA sequencing analysis. A total of 56 faecal samples were collected, including 41 patients with active CD at baseline and 15 of 41 patients providing samples both at week 0 and 24. For subsequent analyses, patients were stratified by week‐24 CDAI into clinical remission (CR; CDAI < 150) and non‐remission (non‐CR; CDAI ≥ 150).

### 2.4 16S rRNA Sequencing

Microbial community genomic DNA was extracted from 56 samples using the E.Z.N.A. Tissue DNA Kit (Omega Bio‐tek, Norcross, GA, U.S.). The hypervariable region V3‐V4 of the bacterial 16S rRNA gene was amplified with primer 338F (5′‐ACTCCTACGGGAGGCAGCAG‐3′) and 806R (5′‐GGACTACHVGGGTWTCTAAT‐3′) using an ABI GeneAmp 9700 PCR thermocycler (ABI, CA, USA). Purified amplicons were pooled in equimolar and paired‐end sequenced on an Illumina MiSeq PE300 platform (Illumina, San Diego, USA). The raw reads were submitted to the NCBI Sequence Read Archive (SRA) database. After initial screening, high‐quality sequences were de‐noised using the unoise3 method by usearch11. The BLAST tool was used to classify all sequences into different taxonomic groups against the SILVA138 database.

### 2.5 Gut Microbiota Analysis

Alpha diversity and beta diversity were determined by QIIME (v1.8.0), and visualisations were generated in R (v3.6.0) based on ASVs information. R (v3.6.0) software was used for bar‐plot diagram analysis. LEfSe analyses were performed with the LEfSe tool. PICRUSt (1.0.029) was used to predict the functional potential of the metagenome based on 16S rRNA data.

### 2.6 Statistical Analysis

The clinical characteristics of participants were analysed using IBM SPSS Statistics software (version 26.0). Quantitative variables were summarised as median (interquartile range, IQR), and comparisons between groups were performed using the Wilcoxon rank‐sum test. Categorical variables were expressed as counts and percentages (*n*, %), and compared using the chi‐square test. *P*‐values less than 0.05 were considered statistically significant. Comparison among groups was performed using analysis of molecular variance. Correlation analysis was conducted using Spearman's correlation.

## 3 Results

### 3.1 Patient Characteristics

A total of 51 patients with active CD treated with UST were included; baseline features are summarised in Table 1. The cohort comprised 31 males (60.8%), with a median age of 31 years (IQR 24–39) and a median disease duration of 36 months (IQR 12–60). UST was initiated as first‐line biologic therapy in 33 (65%) patients and as second‐line therapy in 18 (35%) after exposure to ≥ 1 prior biologic, most commonly infliximab. By the Montreal classification, disease location was L1 (ileal) in 28 (55%), L2 (colonic) in 1 (2%), L3 (ileocolonic) in 21 (41%) and L4 (isolated upper GI) in 1 (2%). Overall, 96% had ileal or ileocolonic disease (L1/L3). Disease behaviour was B1 (non‐stricturing/non‐penetrating) in 39 (76%), B2 (stricturing) in 10 (20%) and B3 (penetrating) in 2 (4%). Active perianal disease was present at baseline in 9 (18%).

**TABLE 1:** Baseline characteristics of patients with active Crohn's disease (*n* = 51).

Variable	Active CD *N* = 51
Male	31 (60.8%)
Age, years	31 (24, 39)
BMI, kg/m^2^	19.8 (18.0, 21.4)
Time from diagnosis to UST initiation, months	36 (12, 60)
WBC, 10^9^/L	6.66 (4.80, 7.52)
Hb, g/L	111 (98, 132)
PLT, 10^9^/L	295 (232, 361)
ALB, g/L	39.3 (34.3, 45.0)
CRP, mg/L	10 (4, 21)
FC, ug/g	161 (92, 377)
ESR, mm/h	29 (14, 51)
Prior use of biologics	
First‐line UST therapy	33 (65%)
Second‐line UST therapy	18 (35%)
Infliximab^a^	17 (94%)
Vedolizumab^a^	1 (6%)
Adalimumab^a^	4 (24%)
Disease location	
Ileum (L1)	28 (55%)
Colon (L2)	1 (2%)
Ileocolon (L3)	21 (41%)
Upper gastrointestinal tract (L4)	1 (2.0%)
Disease behaviour	
Non‐stricturing, non‐penetrating (B1)	39 (76%)
Stricturing (B2)	10 (20%)
Penetrating (B3)	2 (4%)
Active perianal disease	9 (18%)

*Note:* Values are *n* (%), or median (interquartile range).

Abbreviations: ALB, albumin; BMI, body mass index; CD, Crohn's disease; CRP, C‐reactive protein; ESR, erythrocyte sedimentation rate; FC, faecal calprotectin; Hb, haemoglobin; PLT, platelet count; UST, ustekinumab; WBC, white blood cell count.

^a^ Percentages for prior biologics are calculated within the second‐line UST subgroup (*n* = 18).

### 3.2 Effectiveness of UST Therapy in Real‐World

After excluding six patients due to loss to follow‐up (LTFU), the final efficacy analysis was performed on a cohort of 45 patients. At week 24, clinical remission and response were achieved in 21 (46.7%) and 37 (82.2%) of the patients, respectively. Numerically higher remission and response rates were observed with first‐line UST compared to second‐line therapy, although the difference did not reach statistical significance (*p* > 0.05).

### 3.3 Distinct Gut Microbiota Composition Associated With UST Therapy

**FIGURE 1:**
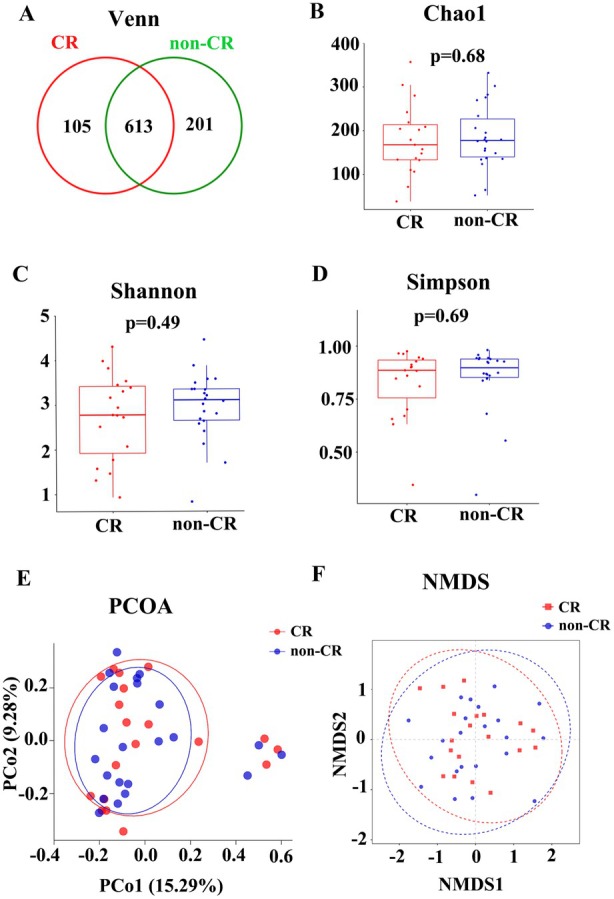
Analysis of baseline gut microbiota diversity of 41 patients (A) Venn diagram of unigenes between CR and non‐CR. (B–D) Alpha diversity indices (Chao1, Shannon, Simpson); no significant differences observed. (E–F) Beta diversity analysis using PCoA and NMDS plots.

**FIGURE 2:**
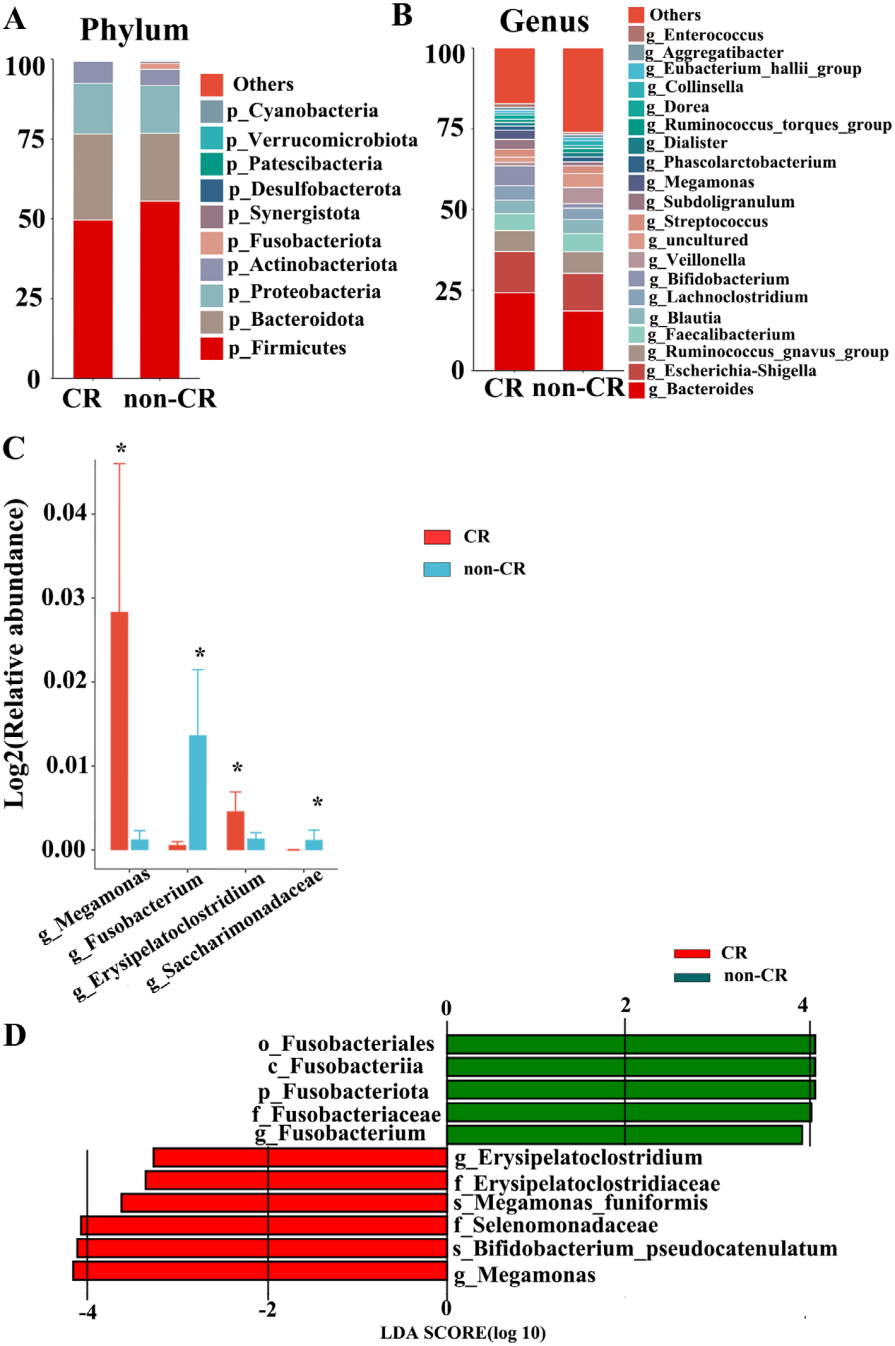
Alterations of baseline gut microbiota composition of 41 patients (A) Bar chart of gut microbiota at the phylum level. (B) Bar chart of gut microbiota at the genus level. (C) Relative abundance of discriminative gut microbiota at the genus level; *p < 0.05. (D) Linear discriminant analysis (LDA) effect size (LEfSe) analysis.

Within the enrolled cohort, baseline faecal 16S rRNA profiles were available for 41 patients and included in the cross‐sectional analysis (CR, *n* = 19; non‐CR, *n* = 22). Baseline clinical characteristics of this 16S subset are summarised in Table S1. A Venn diagram showed 613 shared unigenes between groups, with 105 features unique to CR and 201 unique to non‐CR (Figure 1A). Alpha‐ and beta‐diversity analyses revealed no significant differences between CR and non‐CR (Figure 1B–F). At the phylum level, Fusobacteriota was significantly enriched in non‐CR versus CR (*p* = 0.017; Figure 2A). At the genus/family level, *Megamonas* (*p* = 0.009) and *Erysipelatoclostridium* (*p* = 0.030) were higher in CR, whereas *Fusobacterium* (*p* = 0.016) and Saccharimonadaceae (family; *p* = 0.031) were higher in non‐CR (Figure 2B–C). LEfSe highlighted the enrichment of *Megamonas* and *Erysipelatoclostridium* in CR and *Fusobacterium* in non‐CR (Figure 2D).

### 3.4 Changes in Gut Microbiota During UST Therapy

**FIGURE 3:**
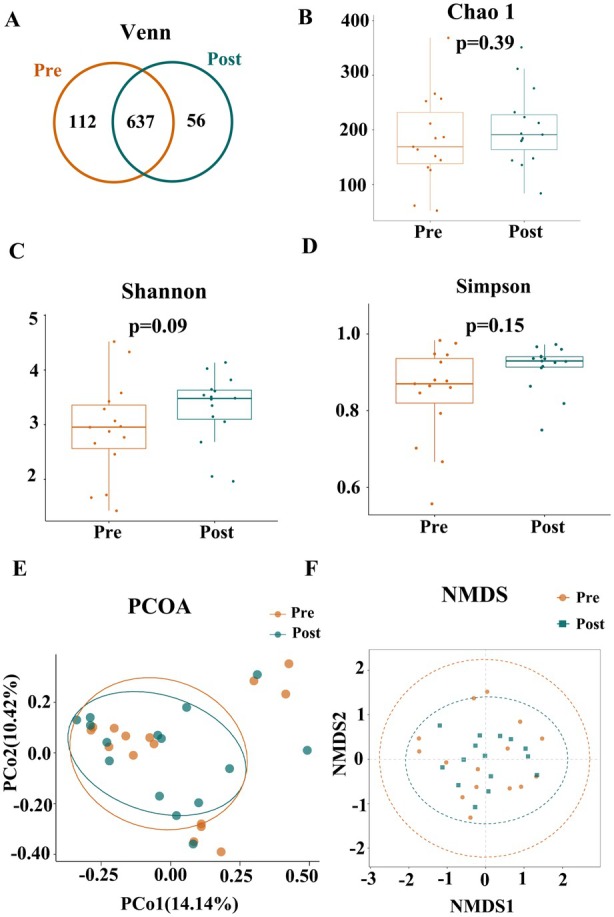
Analysis of gut microbiota diversity of 15 patients (A) Venn diagram of unigenes between pre‐ and post‐treatment groups. (B–D) Alpha diversity indices (Chao1, Shannon, Simpson); no significant differences observed. (E and F) Beta diversity analysis using PCoA and NMDS to visualise overall microbiota structure.

**FIGURE 4:**
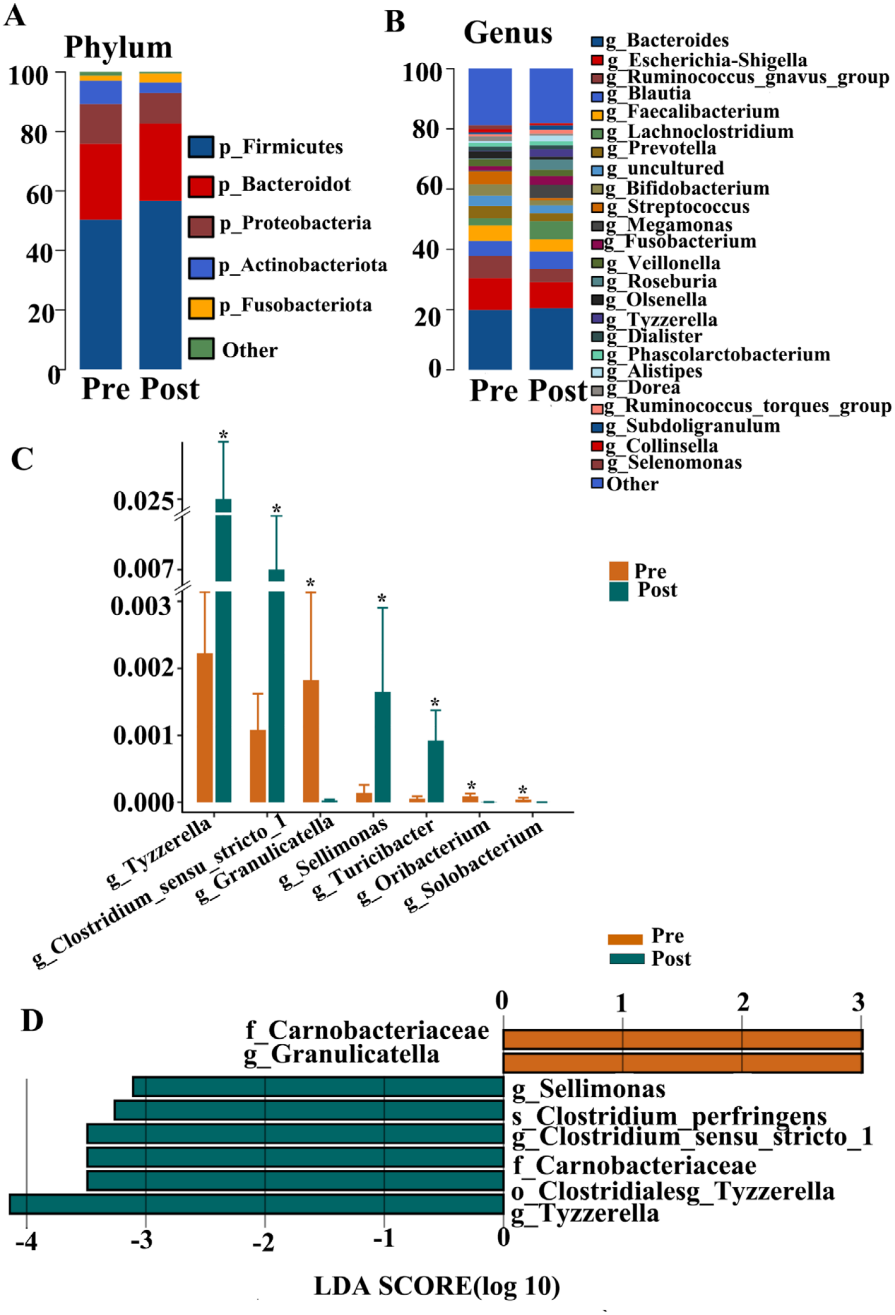
Alterations of gut microbiota composition of 15 patients (A) Bar chart of gut microbiota at the phylum level. (B) Bar chart of gut microbiota at the genus level. (C) Relative abundance of discriminative gut microbiota at the genus level; *p < 0.05. (D) Linear discriminant analysis (LDA) effect size (LEfSe) analysis.

Few longitudinal studies have related gut microbiota to UST treatment response. Paired faecal samples for longitudinal analysis were available from 15 patients at week 0 (Pre) and week 24 (Post). Baseline clinical characteristics of these patients are provided (Table S1). The Venn diagram illustrated 637 shared unigenes between Pre and Post (Figure 3A). Similarly, no significant differences in alpha‐diversity or beta‐diversity were observed between pre‐ and post‐treatment samples (Figure 3B–F). At the phylum level, abundances did not differ between time points (Figure 4A). At the genus level, *Turicibacter* (*p* = 0.020), *Sellimonas* (*p* = 0.033), *Clostridium_sensu_stricto_1* (*p* = 0.045) and *Tyzzerella* (*p* = 0.046) increased from Pre to Post, whereas *Granulicatella* (*p* = 0.013), *Oribacterium* (*p* = 0.027) and *Solobacterium* (*p* = 0.038) decreased (Figure 4B–C). LEfSe confirmed differential abundance of *Tyzzerella*, *Clostridium_sensu_stricto_1*, *Sellimonas* and *Granulicatella* (Figure 4D). In a subgroup analysis by remission status (CR, *n* = 6; non‐CR, *n* = 9), week 0 *Lachnoclostridium* was higher in non‐CR (*p* = 0.042); at week 24, CR patients showed increased *Clostridium_sensu_stricto_1* (*p* = 0.028) and decreased *Granulicatella* (*p* = 0.043).

### 3.5 Prediction of Gut Microbiota Function Associated With UST Therapy

**FIGURE 5:**
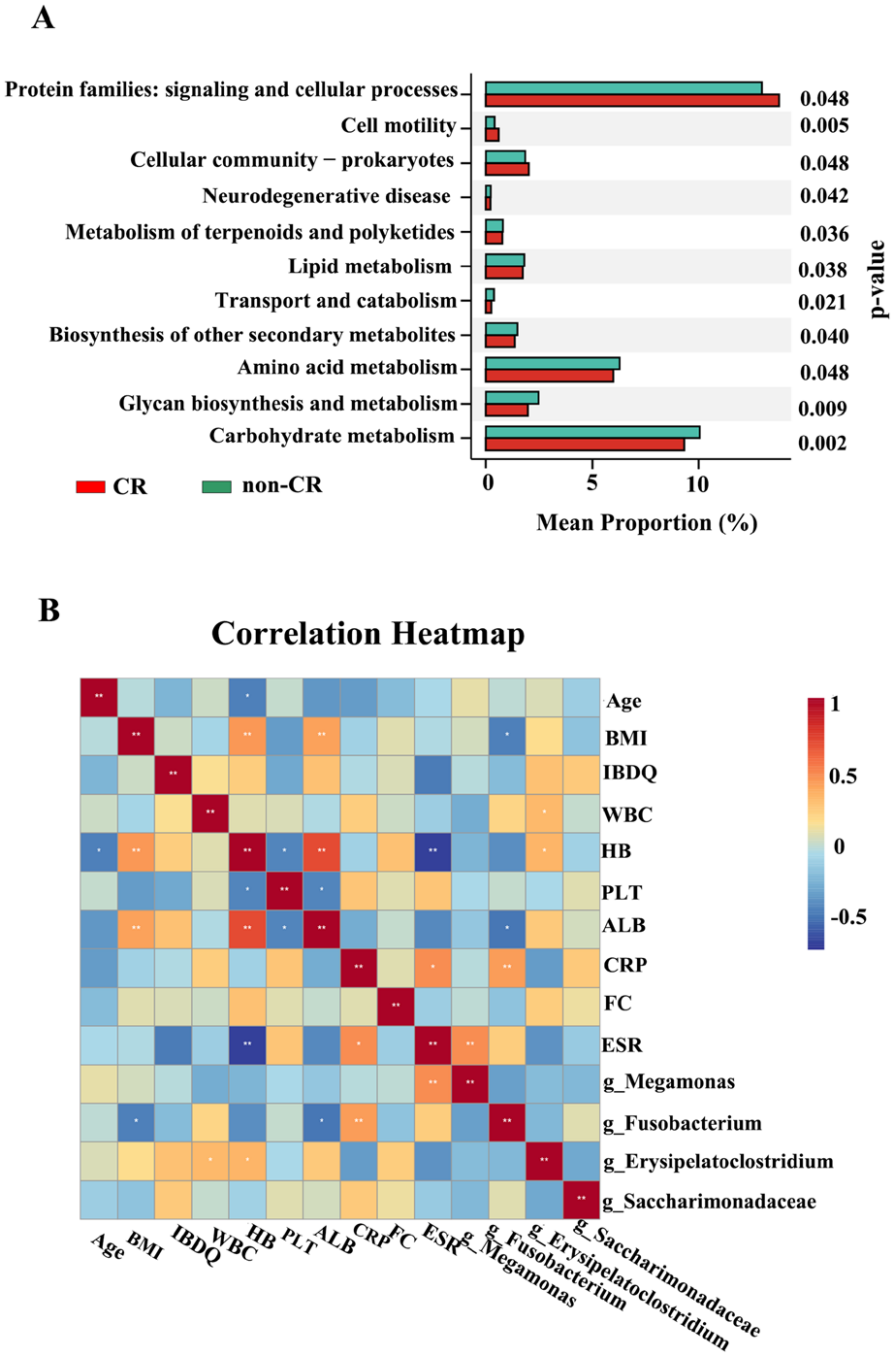
(A) Bar chart of Prediction of gut microbiota function by Phylogenetic Investigation of Communities by Reconstruction of Unobserved States (PICRUSt). (B) Heatmap of differential microbial taxa associated with clinical indicators by Spearman correlation analysis.

Analysis of functional abundance revealed that a significant portion of microbial functions was associated with pathways related to genetic information processing and material metabolism (Figure 5A). Differential enrichment analysis showed that protein families involved in signal transduction and cellular processes, as well as cell motility, were more active in the CR group. In contrast, the non‐CR group was mainly characterised by enrichment of carbohydrate metabolism, glycan biosynthesis and metabolism, and amino acid metabolism.

### 3.6 Correlation Analysis Between Microbial Abundance and Clinical Indicators

Spearman correlation analysis indicated that the abundance of *Fusobacterium* was positively correlated with C‐reactive protein (CRP; *r* = 0.45, *p* = 0.009) and negatively correlated with body mass index (BMI; *r* = −0.35, *p* = 0.015) and serum albumin (ALB; *r* = −0.37, *p* = 0.023). The abundance of *Erysipelatoclostridium* showed a positive correlation with haemoglobin (Hb; *r* = 0.37, *p* = 0.016) and white blood cell count (WBC; *r* = 0.36, *p* = 0.019). The abundance of *Megamonas* exhibited a positive correlation with erythrocyte sedimentation rate (ESR; *r* = 0.50, *p* = 0.008) (Figure 5B).

## 4 Discussion

Our study analysed data from real‐world UST therapy and faecal microbiota in Chinese patients with active CD. The results indicated that the clinical remission rate and clinical response rate of UST therapy were 46.7% and 82.2% at week 24, respectively. Previous medication treatments were not associated with the efficacy of UST. Baseline faecal microbiota sequencing suggested that an increased abundance of *Fusobacterium* was negatively correlated with clinical remission at week 24, as well as positively correlated with CRP and negatively correlated with ALB and BMI, highlighting the role of gut microbiota in treatment response and its potential as a biomarker reservoir.

To our knowledge, this is the first study to explore the relationship between *Fusobacterium* abundance and UST treatment response in a Chinese patient population, thereby contributing novel data from an underrepresented ethnic group.

As a humanised monoclonal antibody, UST has been shown to induce and maintain remission in global clinical trials of CD patients (Sandborn et al. 2022; Kucharzik et al. 2023). However, real‐world evidence demonstrates variable rates of clinical remission and response following UST therapy for CD (Zhou et al. 2023). Differences in clinical efficacy among studies may be attributed to variations in disease severity, trial design, follow‐up duration and treatment protocols. Our findings showed that prior exposure to biologics had no influence on the efficacy of UST, indicating that UST could be an alternative treatment for non‐responders to infliximab or conventional treatment.

Although there is heterogeneity in the relationship between the gut microbiota of CD patients and drug treatment, several studies have found that patients with more diverse and higher microbiota diversity at baseline tend to exhibit more favourable responses to treatment (Zhou et al. 2018). In our study, alpha‐ and beta‐diversity analyses of bacterial communities did not reveal significant differences between the CR and non‐CR groups. However, compared to the non‐CR group, we found an increased abundance of *Megamonas* and *Erysipelatoclostridium* in the CR group. *Megamonas*, a genus first identified in China, is considered a characteristic component of the Asian gut microbiota. It belongs to the Firmicutes phylum and participates in carbohydrate fermentation to produce short‐chain fatty acids (Viladomiu et al. 2017; Jin et al. 2019). As a butyrate‐producing genus, *Megamonas* may promote intestinal barrier integrity by enhancing tight junctions and mucus secretion via AMPK and Akt signalling (Recharla et al. 2023). *Erysipelatoclostridium* was initially identified in patients with colorectal tumours (Wu et al. 2021). It has been reported that *Erysipelatoclostridium* was enriched in post‐treatment colitis mice (Chang et al. 2022), and its abundance correlates positively with serum vitamin D levels in IBD patients (Chen, Li, et al. 2020). We speculate that a higher baseline abundance of *Megamonas* and *Erysipelatoclostridium* may benefit the intestinal mucosal barrier, thereby maintaining gut homeostasis and promoting the clinical efficacy of UST.

*Fusobacterium* was significantly more abundant in the non‐CR group. *Fusobacterium* is a predominant genus within the phylum Fusobacteriota, mainly colonising the oral and colonic mucosa of healthy humans (Quaglio et al. 2022). The notorious *Fusobacterium* species— *Fusobacterium nucleatum* , has been associated with various diseases, including periodontal disease, rheumatoid arthritis, colon cancer and IBD. Numerous studies have reported that *Fusobacterium* was significantly enriched in CD compared to healthy controls (El Mouzan et al. 2018). Interestingly, the strains of *Fusobacterium nucleatum* isolated from the inflamed tissues of IBD are more invasive than strains isolated from healthy tissues (Fan et al. 2023), and its abundance is associated with disease activity (Gao et al. 2023), suggesting that *Fusobacterium nucleatum* may be a potential pathogenic genus (Gevers et al. 2014; Purcell et al. 2018). Mechanistic studies have shown that *Fusobacterium nucleatum* exacerbates colitis by impairing epithelial barrier integrity through downregulation of tight junction proteins. In addition, it induces IL‐8 secretion via the TLR2/ERK pathway, which drives neutrophil recruitment and amplifies mucosal inflammation (Wang et al. 2024). In addition, *Fusobacterium nucleatum* can inhibit autophagy and enhance biofilm formation, facilitating microbial persistence and immune evasion (Yu et al. 2017; Montanari et al. 2025). Collectively, these mechanisms may hinder mucosal healing and reduce the efficacy of biologics such as UST. Faecal microbiota transplantation (FMT) has shown promise as a targeted therapy in refractory CD, achieving long‐term symptom relief and steroid‐free remission in real‐world cohorts (Xiang et al. 2020). Given our finding that *Fusobacterium* enrichment is linked to reduced UST response, FMT may offer a microbiota‐targeted strategy to enhance biologic efficacy in CD patients with gut microbiota dysbiosis.

Furthermore, we identified UST‐associated alterations in the faecal microbiota of 15 CD patients. An enrichment of *Turicibacter*, *Sellimonas*, *Clostridium_sensu_stricto_1* and *Tyzzerella* was seen in the post‐group, while *Granulicatella* was strongly overrepresented in the pre‐group. The genus *Turicibacter* has recently been found to be involved in host lipid metabolism and is associated with dietary fat and changes in body weight (Lynch et al. 2023). It has been predominantly identified in colitis mice, where *Turicibacter* serves as core microbiota that could potentially drive the progression of colitis (Shang et al. 2021; Zhang et al. 2021). Conversely, *Tyzzerella* is profoundly enriched in the ileal mucosa of CD and is also found to be increased in responders to adalimumab treatment (Olaisen et al. 2021; Chen et al. 2022). However, in the CR group, there was an increase in the relative abundance of *Clostridium_sensu_stricto_1* and a decrease in *Granulicatella* in the post‐patients. Experimental models have indicated that *Clostridium_sensu_stricto_1* is increased after drug treatment and is regarded as a short‐chain fatty acid‐producing microorganism (Ma et al. 2022; Guo et al. 2023). It has been shown to benefit intestinal barrier function through producing aromatic amines (Sugiyama et al. 2022). Interestingly, our results also revealed an enrichment of pathways related to signal transduction and cellular processes in CR faecal samples, suggesting that microbial functional activity may contribute to the immunomodulatory effects of UST. *Granulicatella*, also known as nutritionally variant Streptococcus, is a commensal bacterium that parasitises in the oral, intestinal, and urogenital tracts of humans. In vitro experiments involving IBD have demonstrated that decreased *Granulicatella* plays an essential role in maintaining the balance of the gut microbiota (Chen, Chen, et al. 2020). Therefore, regulating gut microbiota composition may exert a positive influence on non‐CR patients.

In summary, distinct differences in gut microbiota composition and predicted function were observed between patients who achieved clinical remission and those who did not. Notably, a higher baseline abundance of *Fusobacterium* was associated with reduced UST response, suggesting a potential role of this genus as a predictive biomarker.

However, this study has several limitations. First, it was a single‐center exploratory cohort with a relatively small sample size and limited longitudinal follow‐up (*n* = 15), which may reduce statistical power and generalisability. Second, key confounding factors—such as diet, recent antibiotic use and disease severity—were not fully accounted for due to practical constraints. Third, microbiota analysis was based on 16S rRNA sequencing without metagenomic, metabolomic or strain‐level validation. Future studies incorporating larger, multi‐center cohorts and multi‐omics approaches are warranted to validate these findings and further elucidate the mechanistic links between microbial dysbiosis and UST treatment response in CD.

## 5 Conclusion

Our study demonstrated the significant clinical efficacy of UST in treating active CD. Moreover, elevated baseline *Fusobacterium* abundance was found to be associated with poorer UST response, highlighting its potential as a predictive biomarker of treatment outcomes in CD. These findings underscore the potential role of gut microbiota in shaping treatment outcomes and support further investigation into microbiota‐based therapeutic strategies for CD.

## Author Contributions

**Chengran Wang:** writing – original draft, conceptualization, methodology, investigation. **Yanping Hao:** conceptualization, investigation, writing – original draft, methodology. **Yunyun Liu:** conceptualization, investigation, writing – original draft, methodology. **Le He:** software, data curation. **Su Xu:** software, data curation. **Minna Zhang:** resources, visualization. **Xin Wang:** writing – review and editing, project administration. **Honggang Wang:** writing – review and editing, project administration.

## Conflicts of Interest

The authors declare no conflicts of interest.

## Supporting information


**Table S1:** Baseline Clinical Characteristics of Patients in the CR and non‐CR Groups.

## Data Availability

The data that support the findings of this study are available on request from the corresponding author. The data are not publicly available due to privacy or ethical restrictions.
