# Supplementary material for: Baseline Fusobacterium Abundance Predicts Ustekinumab Response in Crohn's Disease: A Prospective Microbiome Cohort Study
Source: Microb Biotechnol. 2025 Oct 24;18(10):e70250. doi: 10.1111/1751-7915.70250 (PMC12551668; doi:10.1111/1751-7915.70250)
Supplement: Supplementary file 1 — Table S1: Baseline Clinical Characteristics of Patients in the CR and non‐CR Groups. [file MBT2-18-e70250-s001.docx]

Table S1. Baseline Clinical Characteristics of Patients in the CR and non-CR Groups

| Variable | CR group | non-CR group | p-value |
| --- | --- | --- | --- |
| Age, years | 35.50 (33.25, 40.00) | 33.00 (29.00, 36.00) | 0.442 |
| Male | 4 (66.7%) | 7 (77.8%) | 1.000 |
| BMI,Kg/m2 | 20.29 (19.32, 21.57) | 19.28 (15.92, 21.44) | 0.529 |
| WBC, 10^9/L | 4.70 (4.11, 6.19) | 6.62 (6.25, 7.38) | 0.224 |
| Hb, g/L | 126.00 (104.00, 142.75) | 107.00 (89.00, 125.00) | 0.272 |
| PLT, 10^9/L | 237.00 (186.75, 293.25) | 318.00 (292.00, 355.00) | 0.145 |
| ALB, g/L | 44.25 (43.05, 45.30) | 37.35 (32.75, 42.53) | 0.070 |
| CRP, mg/L | 3.78 (3.34, 4.49) | 17.35 (9.79, 30.43) | 0.033* |
| FC, ug/g | 319.40 (224.35, 414.45) | 292.60 (99.40, 833.90) | 0.889 |
| ESR, mm/h | 10.00 (7.50, 12.50) | 39.00 (25.00, 53.75) | 0.038* |

Values are presented as n (%) for categorical variables and median (interquartile range) for continuous variables.

Abbreviations: WBC, white blood cell count; Hb, hemoglobin; PLT, platelet count; ALB, albumin; CRP, C-reactive protein; FC, fecal calprotectin; ESR, erythrocyte sedimentation rate.
